# Supplementary material for: Empowering Young Women: A Qualitative Co-Design Study of a Social Media Health Promotion Programme
Source: Nutrients. 2024 Mar 9;16(6):780. doi: 10.3390/nu16060780 (PMC10975755; doi:10.3390/nu16060780)

THE DAILY HEALTH COACH CO-DESIGN CONCEPT MAP

Research Question One

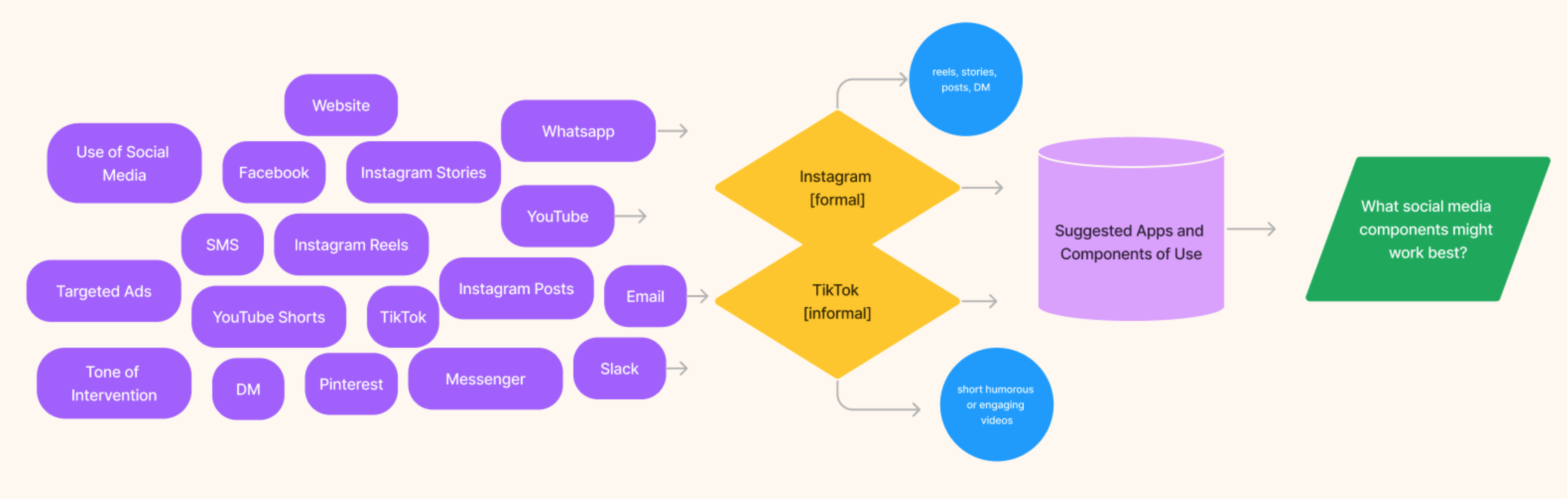

THE DAILY HEALTH COACH CO-DESIGN CONCEPT MAP

Research Question Two

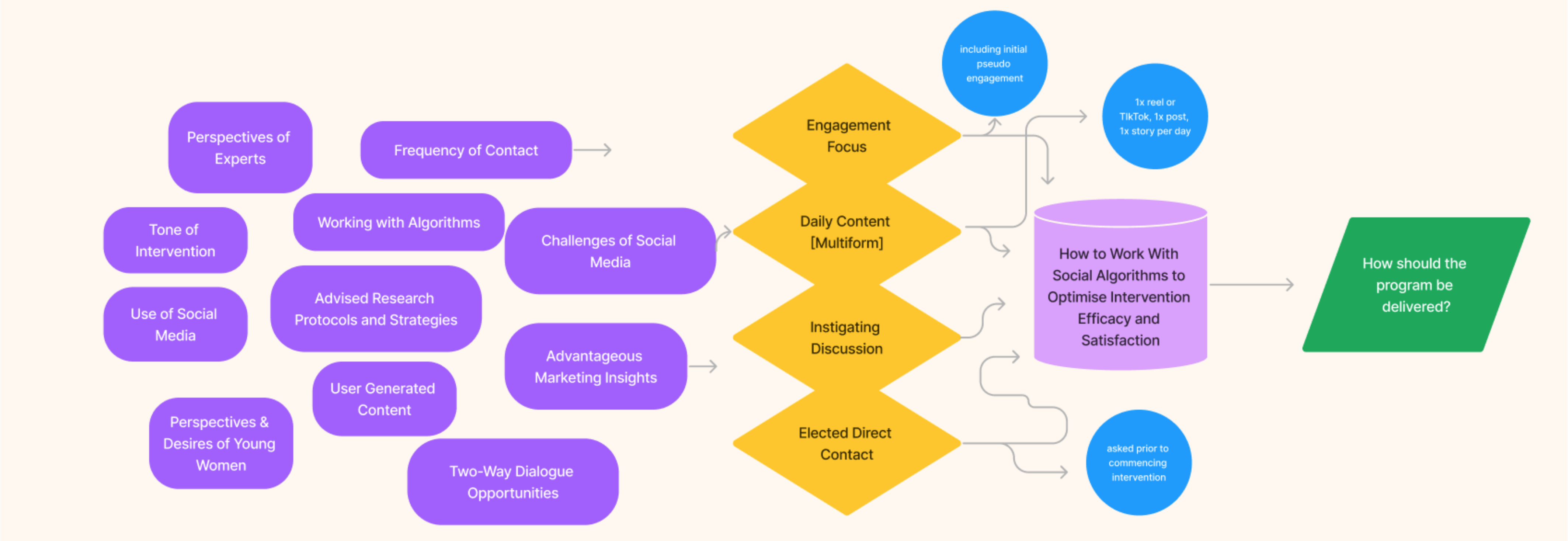

Research Question Three

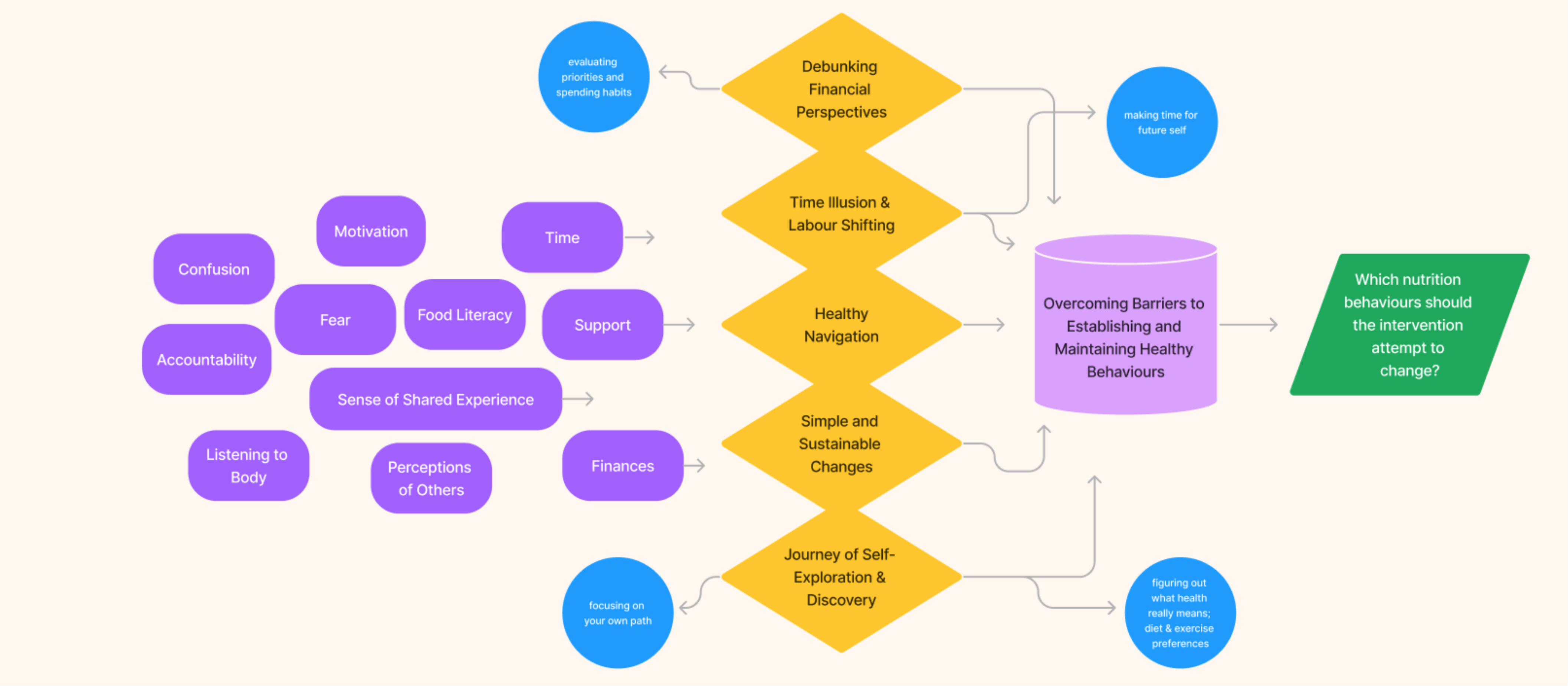

Research Question Three

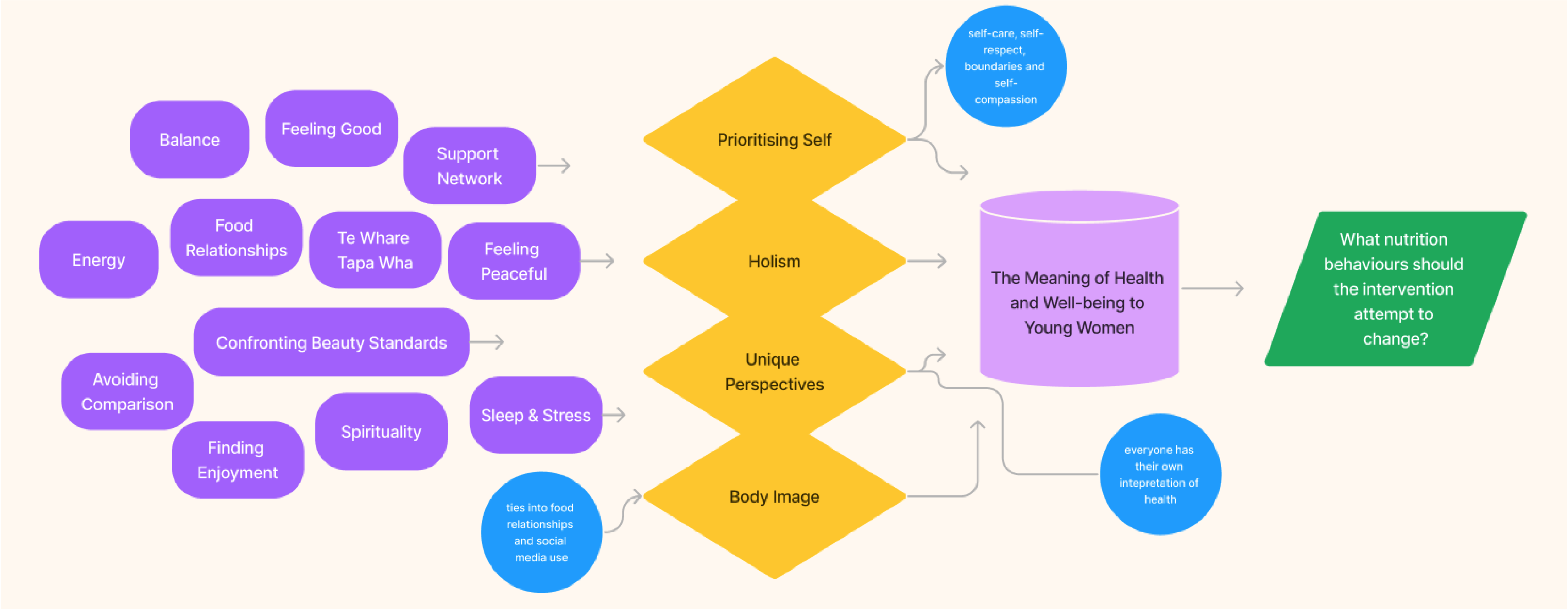

Research Question Four

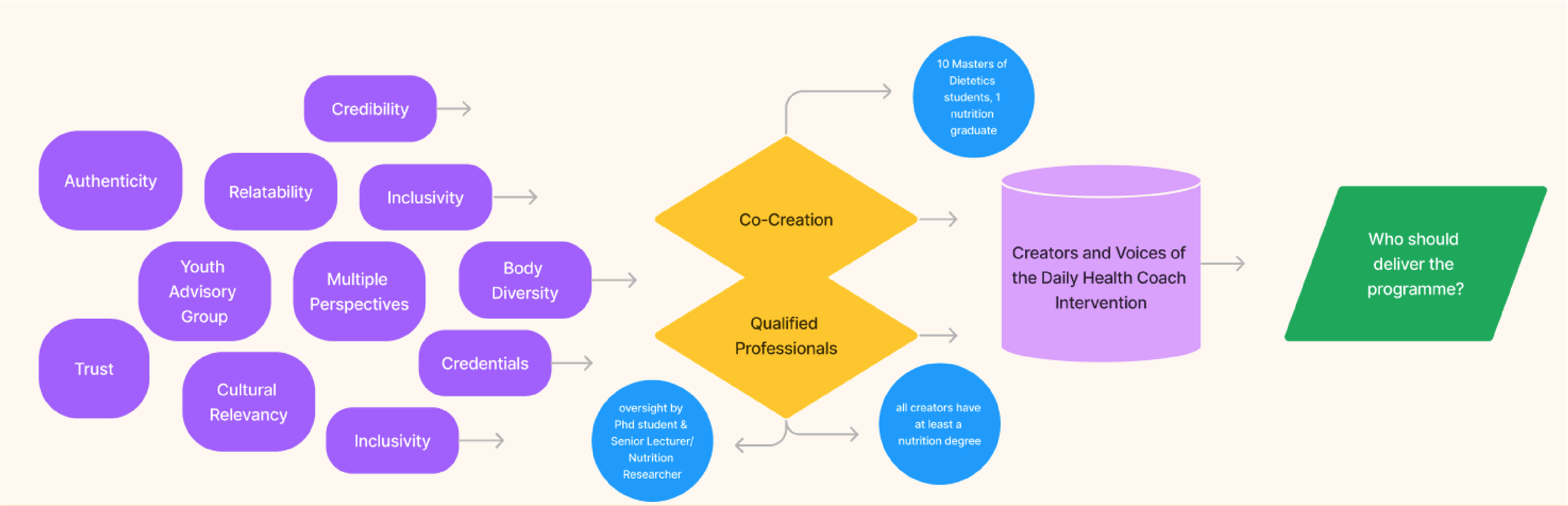

Legend

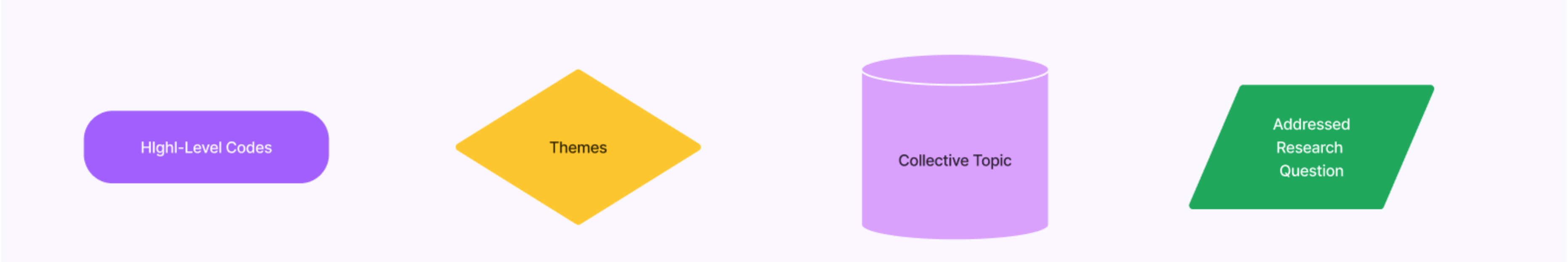

Supplement: Supplementary file 1 [file nutrients-16-00780-s001.zip › nutrients-2895731-supplementary.pdf]
